# Reality

**Published:** 1885-07

**Authors:** J. B. Chandler


### REALITY.

J. B. CHANDLER.

It is not improbable that we are far from realizing the reality of our existence.

The man who can look back over a period of three score years can tell you he remembers when all was swamp and woods where now are large cities, with hundreds of thousand inhabitants. Can tell you when the city of New York was almost confined within the old cowpaths between the East and North Rivers and City Hall and the Battery. When the old City Hotel (long since destroyed) was the great resort for travelers, until superseded by the then wonderful palatial Astor House. When Philadelphia was confined within a narrow strip from Broad street to the Delaware and from South street to Vine, with some outlying districts more or less built up—having separate organizations and governments. When Chicago was not thought of and the large cities of the far west were the hunting grounds of Indian tribes now almost extinct.

The effect of time upon events in which we participated, aided by the pressure of new lights, new discoveries, new adaptations, seems to prevent us from actually realizing the changes or at least makes dim the realization that any other state of affairs ever existed.

We jump into an express train in the evening, go quietly to sleep in a comfortable bed, awake the next morning at our journey's end, four or five hundred miles from home; do we realize that, easily within our recollection, such a trip under most favorable circumstances would have involved the fatigue and discomfort of a stage ride of four or five days continuous journey? We step into a Telephone station, call up a friend fifty or one hundred miles away and listen to his familiar voice, complain probably of a little interference by induction from other wires and batteries, but, all the same, we understand our friend and he us; which a few years ago would not only have been considered an impossibility, but the man who suggested such a result would have been thought a candidate for lunacy. We do this but do we realize the change and its consequences?

We leave a "call order" at an express office; the next morning our package is delivered to a friend hundreds of miles away. Do we remember that in our time the means of transit for such a package was the old Conestoga wagon which after a day or two spent in loading and stowing its freight lumbered along over the hills two or three miles an hour?

The "fast" sailing line of packets for Liverpool that sometimes made trips from Liverpool to New York or Philadelphia in thirty days, but frequently in sixty and were the only means of communication by which we received the important commercial and other advices from Europe. Do we realize the change now when we actually receive messages from there by telegraph, which, apparently, from difference of longitude and their almost instantaneous transmission, arrive here *hours before they are sent?*

The steel and flint have been laid aside for matches, the penny dips, given place to the brilliant oil lamps, and gas in many places is superseded by electric lights. Wood fires are comparatively no more, whilst anthracite for heating and cooking purposes is likely soon to be abandoned for water gas or, where obtainable, natural gas.

In the manufacture of gas from water and its adaptation for both lighting and heating, great strides have been made within a short time. It was the privilege of the writer within a few weeks to be present at a meeting of experts from various parts of the country, to inspect some methods of manufacturing the gas, carbureting it for illuminating purposes, and utilizing it for heating and cooking without carbureting. We sat in a room in which a stove was put up to do the cooking for a very delicious dinner, which we witnessed cooked and of which we ate heartily. Amongst the delicacies was a planked shad; the heat was reflected upon it from fire brick through interstices in which the burning gas permeated creating a heat entirely within the control of the attendant cook; the result was a specimen of the culinary art perfect throughout in delicate brown hew and retaining all its juices. Indeed it was claimed that mutton chops of which we had a specimen, instead of losing weight in cooking actually gained from retention of juices and absorption.

A loaf of bread was sliced and toasted both sides in three minutes perfectly, nicely browned and no burnt spots. The table was lighted brilliantly, by two burners (two feet per hour each) of water gas uncarbureted, injected into the flame of an ordinary coal oil lamp. Again, we had specimens of the gas carbureted by the process of passing through vapor from coal oil.

The crowning triumph of the evening however was the light produced by the uncarbureted gas, burning in connection with an arched wire of some material (not platina) said to be indestructible; the gas is delivered through an ordinary bat-wing burner over which the wire is placed just high enough to become incandescent from the burning blue gas, the result with the accompanying reflector is the most steady light I have ever witnessed. By an ingenious combination of glass dome and drums this light is made to do double duty of both lighting and heating a large room; the products of combustion are carried away by invisible communication with a flue, and the whole apparatus is an artistic ornament to the parlor. Water gas can now be made for probably one half the cost of coal gas. Coal fires, ashes and dirt, dried-up meats, burnt toast must soon take their places in the past and we shall gradually drift into the change without realizing it.

It would take volumes to chronicle the wonderful discoveries of science and art which have been crowded into the short period embraced within the past sixty years. Under the researches of scientific and ingenious men the laws of forces have been, through wonderful machinery, made almost submissive to the will of man. Space has been almost annihilated; what seemed insurmountable natural barriers have yielded to the combination of engineering skill and willing capital. The peoples of the world have been brought in closer communion.

The labors of the press, powerful for good or evil are not to be overlooked in the contemplation of the recent past or in prognostications for the future. It is within easy recollection when a circulation of one or two thousand copies was large for a daily paper and indeed was all that was practicable for the then crude and slow means of printing. The character of those old newspapers could only compare in favorable light morally, with their wonderful successors of the present.

The daily history of the world is now recorded for the subscriber's morning reading, whilst a dishing up of petty local items and some long-on-the-way news from other points domestic or foreign made up the enterprising sheet of the past.

Do we realize the change and its consequences? Does any one of us ever stop to consider whether the community is to be benefited by the power of the press?

Our opinions will make no difference; the press will go on and its readers will increase. People will continue to be deceived into the idea that a man because he is the editor of a newspaper cannot and will not lie. That the entire purpose of the press is to benefit mankind and not a means of money making for its proprietors. That the proprietor of some scurrilous sheet who parades as a great public benefactor in nosing out all dirty scandals and filthy trials and publishing them, is not a pest to society who pandering to a low taste fills his pockets. That, indeed, what our morning paper says *must* be true because it is the advocate of our party or of our church. Do we realize this great power which is the mouth piece of every good and every bad? Which carries into almost every family daily, all accounts of murders, thefts, Anarchist and Socialist vaporings mingled with church news and the doings of the kingdoms of the world.

Again: do we realize the moral changes of the past three score years? The struggle of the people for relief from old burdens of oppression —African slavery has been wiped out from almost every country except perhaps Africa itself—We have not yet realized the cost of its extirpation here, nor the real benefits which must ultimately result.

Europeans are moving onward in the march of improvement. Italy has thrown off the shackels that held her people in almost bondage.

A great war has been fought between France and Germany; its instigators were defeated. Who knows the real cause of that war? And when shall we realize its effect?

This country is hastening, through natural increase and immigration, toward a full population. From all classes in Europe extending through the vilest up to the best we are making new citizens. They bring with them the habits, prejudices and vices of their old homes. Our untramelled freedom is a boon they seek and many use it to the disadvantage of their adopted country.

Do we realize that all the discoveries, improvements, changes of population, etc., are crowded within such a small space of time, that it is not less wonderful that people have been able to adapt themselves to the sudden changes; yet so far, we have avoided any great convulsions.

Elements that may produce temporary trouble are busy struggling up for power ; the leven which has kept order in this country so far is fast becoming a minority, Our imported citizens if they excel in any one thing more than another, it is their unrestrained procreative inclinations, whilst their families are large and the swarms therefrom early and frequent, prudence and perhaps natural causes contribute to confine within limited number those who are "native and to the manor born." It is not hard to calculate how long it will take in such a state of affairs to change the balance of power. Already those who have ruled abroad are busy trying to keep the children of the immigrants from being absorbed into the general population, they are to be kept *distinct* by religious prejudices and laws. The effort extends from the pauper orphan asylum through every channel that can be reached. It is not an effort to christianize and harmonize but to separate and control. Do we realize what unchecked will result from this? Do we recognize our country as fast becoming the seat of institutions which are being driven out from other countries where they have been tried and found wanting?

Do we realize that the doctrine of "Evolution" is being preached now from the pulpit? And that it is made in capable hands an adjunct of Christianity?

Let us not be pessimists, the age we live in is progressive; the people of this country and indeed of the world are fast becoming more enlightened and when they *do* realize that they are better or worse for anything, they will readily and easily adopt or destroy it. Mistakes may and will be made, unprincipled schemers will obtain a following which may cause trouble, but the world moves onward.

The sexagenarian will realize speedily the great problem involved in death. Whilst the babe of to-day will hurry through the next six decades to probably look back upon more wonderful changes than those of the past.

